# Prevalence and molecular characterization of *Toxoplasma gondii* DNA in retail fresh meats in Canada

**DOI:** 10.1016/j.fawpar.2018.e00031

**Published:** 2018-11-08

**Authors:** Asma Iqbal, Nicol Janecko, Frank Pollari, Brent Dixon

**Affiliations:** a Bureau of Microbial Hazards, Food Directorate, Health Canada, 251 Sir Frederick Banting Driveway, Ottawa, ON K1A 0K9, Canada; b Centre for Food-borne, Environmental and Zoonotic Infectious Diseases, Public Health Agency of Canada, 255 Woodlawn Road West, Unit 120, Guelph, ON N1H 8J1, Canada

**Keywords:** *Toxoplasma gondii*, Prevalence, Genotypes, Meat, Canada

## Abstract

*Toxoplasma gondii* is a protozoan parasite which infects a wide variety of mammals and birds worldwide, including humans. Human toxoplasmosis is primarily transmitted through the ingestion of tissue cysts in raw or poorly cooked meat and organs of infected animals, or through the ingestion of oocysts in food, water or soil contaminated with cat faeces. There is a distinct paucity of information on the prevalence and molecular characteristics of *T. gondii* in retail meats in Canada. This study reports the presence of *T. gondii* DNA in 4.3% (12 of 281) of packages of fresh ground beef, chicken breasts and ground pork purchased at retail in three Canadian provinces. *T. gondii* prevalence was very similar among all three meat types tested, and among the provinces sampled. Genotyping of positive samples by means of PCR-RFLP and DNA sequencing demonstrated the presence of both *T. gondii* type II (66.7%) and type III (33.3%). These findings provide baseline data on the prevalence of *T. gondii* DNA in fresh meats purchased at retail in Canada and will allow for more accurate and meaningful health risk assessments for the purposes of developing food safety guidelines and policies.

## Highlights

• *T. gondii* DNA detected in 4.3% (*n* = 281) of packages of beef, chicken and pork in Canada.
• *T. gondii* prevalence was similar among the meat types tested and provinces sampled.
• Genotyping of positive samples demonstrated both *T. gondii* type II and type III.

## 1 Introduction

*Toxoplasma gondii* is an obligate intracellular protozoan parasite found worldwide in virtually all mammals and birds (Reviewed in Hill et al., 2005; Dubey, 2010). Cats and other felids represent the only definitive hosts in the life cycle of *T. gondii*, and are responsible for shedding oocysts into the environment which then become infectious to intermediate hosts, including humans and meat animals (Reviewed in Pereira et al., 2010). While humans may become infected with *T. gondii* through the ingestion of oocysts in food, soil or water, transmission also commonly occurs through the ingestion of tissue cysts in raw or inadequately cooked meats. Raw or undercooked meat has been reported to be a risk for *T. gondii* infections in numerous outbreak investigations and epidemiological studies (Reviewed in Jones and Dubey, 2012).

Infections with *T. gondii* are generally asymptomatic in humans, although mild flu-like symptoms are not uncommon (Reviewed in Dubey, 2010). Immunocompromised individuals may develop severe symptoms including encephalitis, myocarditis and pneumonia. Toxoplasmosis of the brain is an AIDS-defining illness, and a leading cause of death. Congenitally infected infants may also be severely affected (e.g., intellectual disability, hydrocephalus, retinochoroiditis, hepatosplenomegaly).

Infection with *T. gondii* is one of the leading causes of food-borne illness hospitalizations, representing 8% of the total number in the U.S. (Scallan et al., 2011). It is also the second highest cause of death (24% of total deaths) due to food-borne illness after non-typhoidal *Salmonella* spp. (Batz et al., 2011). The annual cost of illness due to infection with *T. gondii* in the U.S. has been estimated to be close to $3 billion (Batz et al., 2011), and the pathogen-food combination of *Toxoplasma* and pork has been ranked second only to *Campylobacter*-poultry in terms of annual food-borne disease burden in the U.S. (Batz et al., 2011). *Toxoplasma*-beef was also in the top ten pathogen-food combinations in the same study.

In North America and Europe, most *T. gondii* isolates have been classified into three clonal lineages or genotypes (type I, type II and type III) (Howe and Sibley, 1995; Su et al., 2006). Although it remains unclear to what extent the variability in the severity of symptoms is associated with *T. gondii* genotypes, type II isolates have been found in >70% of cases of human toxoplasmosis (Howe and Sibley, 1995; Howe et al., 1997; Ajzenberg et al., 2002).

Numerous studies have reported on the prevalence of *T. gondii* in meat animals (Reviewed in Dubey, 2010; Belluco et al., 2016), although a high degree of variability exists in reported prevalences depending upon the age and strain of the animals, the location of the study and the type of serological test used. Based on high seroprevalence, which exceeds 10–20% in most countries, pigs may be the most important meat animal in the transmission of infection to humans (reviewed in Dixon et al., 2011). Infection in cattle is generally less prevalent than in pigs in most regions (Reviewed in Macpherson et al., 2000). This may be due to an apparent resistance to *T. gondii* in cattle (Reviewed in Fayer, 1981). The role of cattle in the transmission of toxoplasmosis is, however, still unknown (reviewed in Jones and Dubey, 2012). Relatively few studies have been done on the prevalence of *T. gondii* in chickens (Reviewed in Guo et al., 2015). However, a recent study by Ying et al. (2017) reported a relatively high seroprevalence of 19.4% in free-range chickens from grocery stores and farms in three states in the U.S.

Studies have also been done worldwide to determine the prevalence of *T. gondii* in retail meats (Reviewed in Guo et al., 2015). Mouse and cat bioassays are the gold standards for detecting the presence of *T. gondii* tissue cysts in meats (Reviewed in Dixon et al., 2011; Guo et al., 2015). In addition to being very sensitive, these methods also allow for the determination of viability of the tissue cysts and are, therefore, very useful in terms of risk assessment. Furthermore, genotyping or complete DNA sequence analyses can be performed by extracting DNA from mouse tissues and performing PCR to amplify *T. gondii* DNA. Finally, mouse bioassays also allow for the acquisition of *T. gondii* isolates for further research efforts. However, bioassays are expensive, time-consuming and have ethical considerations. As a result, many studies on *T. gondii* in meats have made use of PCR-based methods, or serological assays (e.g., MAT, IFAT, ELISA), which are considerably faster and cheaper. For example, Opsteegh et al. (2010) developed a magnetic capture PCR for the detection of *T. gondii* in meat samples of up to 100 g. Another benefit in using PCR assays is that DNA sequencing or restriction fragment length polymorphism (RFLP) analyses can be performed in order to confirm the presence of *T. gondii*, and to determine the genotype.

Due to the paucity of information on the prevalence and the molecular characteristics of *T. gondii* in retail meats in Canada it is difficult to estimate the risk to consumers or to develop meaningful health risk assessments for the purpose of developing food safety guidelines and policies. As chicken, beef, and pork represent the most commonly consumed meats in Canada (Agriculture and Agri-Food Canada, 2018), the objective of the present study was to establish a baseline prevalence of *T. gondii* DNA in these retail fresh meats, as well as to identify the predominant genotypes.

## 2 Materials and methods

### 2.1 Retail meat samples

From April 2015 to December 2015, a total of 281 packages of retail fresh meat (93 packages of ground beef, 94 packages of chicken breasts, and 94 packages of ground pork) were purchased at grocery stores in three Canadian provinces (British Columbia, Alberta and Ontario) by FoodNet Canada (Public Health Agency of Canada). Whenever it was listed on the package label, the origin of the meat tested in this study was always Canada. Utilizing the FoodNet Canada sample collection framework, approximately 9 packages (range 6–12) including all three meat types were purchased and shipped by overnight courier to the Parasitology Laboratory, Bureau of Microbial Hazards, Health Canada, in Ottawa, in coolers containing ice packs and temperature loggers. Upon receipt, the packages were immediately transferred to a 4 °C refrigerator and stored for a maximum of 24 h until sample processing could be done, or frozen at −20 °C if processing could not be done within 24 h of receipt.

### 2.2 Sample processing

For ground beef and ground pork, each package was tested as a single sample. Since retail packages of chicken breasts generally contain multiple pieces, each chicken breast was tested individually and results were recorded for both the entire package and for the individual chicken breasts (*n* = 234). Approximately 200 mg of meat was excised from the interior of each chicken breast, and removed from the interior of the ground beef and pork, using sterile instruments.

### 2.3 DNA extraction

Each portion of meat was immediately frozen in liquid nitrogen and pulverized with a cleaned and sterilized mortar and pestle. For the molecular characterization of *T. gondii* isolates, DNA was extracted from all pulverized samples using Easy-DNA™ Kit (Invitrogen, cat. # K1800–01) according to the manufacturer's protocol (#3). DNA templates were stored at −20 °C until analyzed. In order to minimize any DNA contamination, a room separate from the PCR amplification laboratory was used for all DNA extractions, and swabs from various working areas were tested. Along with the meat samples, DNA was simultaneously extracted from *T. gondii*-positive (type II) and negative tissues from lab stock samples (beluga) (Iqbal et al., 2018). These extraction controls were included in all PCR reactions and on the subsequent gels.

### 2.4 Polymerase chain reactions

Screening of retail fresh meat samples for the presence of *T. gondii* DNA involved PCR amplification of the 529-bp fragment, as well as a fragment of the B1 gene (Table 1). Meat samples were considered to be PCR-positive for *T. gondii* if one or both of these genes amplified. To determine the genotypes of *T. gondii* present, multilocus genotyping, consisting of nested-PCR on four different restriction fragment length polymorphism (RFLP) markers: SAG2 3′, SAG2 5′, GRA6 and BTUB genes, was used (Table 1).

**Table 1:** Primers and restriction enzymes used for nested-PCR and RFLP on *Toxoplasma gondii* in retail fresh meat packages in Canada.

Genetic markers	External primers (5′-3′)	Internal primers (5′-3′)	Restriction enzymes	Reference
529-bp	Tox4: CGCTGCAGGGAGGAAGACGAAAGTTG Tox5: CGCTGCAGACACAGTGCATCTGGATT	NA^a^	NA	Homan et al. (2000)
B1	OutF: GGAACTGCATCCGTTCATGAG OutR: TCTTTAAAGCGTTCGTGGTC	IntF: TGCATAGGTTGCAGTCACTG IntR: GGCGACCAATCTGCGAATACACC	NA	Di Guardo et al. (2011)
BTUB	ExtF: TCCAAAATGAGAGAAATCGT ExtR: AAATTGAAATGACGGAAGAA	F: GAGGTCATCTCGGACGAACA R: TTGTAGGAACACCCGGACGC	*Bsi*EI; TaqI	Zhou et al. (2013); Khan et al. (2005)
GRA6	FO: GGCAAACAAAACGAAGTG RO: CGACTACAAGACATAGAGTG	F: GTAGCGTGCTTGTTGGCGAC R: TACAAGACATAGAGTGCCCC	*Mse*I	Fazaeli et al. (2000); Zakimi et al. (2006)
SAG2 5′	F4: GCTACCTCGAACAGGAACAC R4: GCATCAACAGTCTTCGTTGC	F: GAAATGTTTCAGGTTGCTGC R2: GCAAGAGCGAACTTGAACAC	Sau3IA	Howe et al. (1997)
SAG2 3′	F3: TCTGTTCTCCGAAGTGACTCC R3: TCAAAGCGTGCATTATCGC	F2: ATTCTCATGCCTCCGCTTC R: AACGTTTCACGAAGGCACAC	*Hha*I	Howe et al. (1997)

a NA = not applicable.

The quality and banding intensity of individual PCR amplicons was examined on 1.5% agarose gels containing GelRed (0.5 μg/ml). As a reference for molecular size, a 100-bp DNA ladder (Fermentas, cat. # SM0241, ON, Canada) was diluted to give a final concentration of 0.5 μg/10 μl in TE buffer (10 mM Tris-HCl, 1 mM EDTA, pH 8.0) and 1× loading buffer. From the prepared mixture, 5 μl was added to the well designated as reference.

Positive control (extracted DNA from goose brain experimentally infected with *T. gondii* type III) received from Dr. Emily Jenkins, Department of Veterinary Microbiology, University of Saskatchewan, and negative control (DNase-free water instead of DNA template), were run along with the samples on each PCR amplification as well as on RFLP gels.

#### 2.4.1 Amplification of 529-bp fragment

The 529-bp fragment was amplified according to Homan et al. (2000), using a 26-mer forward primer (Tox4) and 26-mer reverse primer (Tox5) (Table 1). The master mix (50 μl) included 1× PCR buffer, 2.5 mM of MgCl_2_, 200 μM of dNTPs, 0.5 μM of both the forward and reverse primers, 2.5 U of GoTaq polymerase (Promega, cat. # M3005, Wisconsin, USA) and 2 μl of DNA extract.

#### 2.4.2 Amplification of B1 gene

A region of approximately 95 bp within the B1 gene was amplified according to Di Guardo et al. (2011) using the primer pair B1-OutF and B1-OutR in a first round of PCR, followed by a second round using the primer set B1-IntF and B1-IntR (Table 1). Both rounds of the nested-PCR were prepared in a 50 μl master mix containing 1× Flexi buffer, 200 μM of dNTPs, 1.5 mM of MgCl_2_, 1 μM of both the forward and reverse primers, 1.25 U Hot start Taq polymerase (Promega, cat. # M5005, Wisconsin, USA), and adding 2 μl of DNA extract for the first round or 4 μl of round one product for the second round.

#### 2.4.3 Amplification of BTUB gene

Genotyping using the beta-tubulin (BTUB) gene of *T. gondii* was performed by PCR amplification according to Zhou et al. (2013) and Khan et al. (2005). The initial round of amplification with the external primers BTUB-ExtF and BTUB-ExtR (Table 1) was carried out in 25 μl of mixture containing 1× Flexi Buffer, 200 μM of dNTP, 1.5 mM of MgCl_2_, 0.4 μM of forward and reverse primers, 1.25 U of Hot Start Taq polymerase (Promega, cat. # M5005, Wisconsin, USA) and 2 μl of DNA. The first round also included 1.5 μg of bovine serum albumin (BSA) (New England BioLabs, Ipswich, MA). PCR products were diluted (1:10) and used for a second round of amplification with the internal primers BTUB-F and BTUB-R in a 25 μl volume mixture containing the same PCR reagent concentrations described previously, with the exception that 1.5 mM MgCl_2_ was added, and no BSA was used in the second round PCR mixture. The second round amplification protocol was 94 °C for 4 min followed by 30 cycles of 94 °C for 30 s, 60 °C for 1 min, and 72 °C for 2 min, with an over-extension step at 72 °C for 5 min.

#### 2.4.4 Amplification of GRA6 fragment

Nested-PCR was performed to amplify the coding region of the GRA6 gene according to Zakimi et al. (2006), and to verify the presence of *T. gondii* DNA in the meat samples. PCR amplification was performed with 2 μl of DNA template in a 50 μl reaction mixture containing 5× green GoTaq Flexi buffer (Promega, cat. # M891A, Wisconsin, USA), 2.0 mM MgCl_2_ (Promega, cat. # M890A, Wisconsin, USA), 200 μM of each of the four deoxynucleotide triphosphates (dNTP) (Promega, cat. # C1141, Wisconsin, USA), 50 pmol of each primer (Sigma, Oakville, ON), and 1.25 U of Go Taq Hot Start Polymerase (Promega, cat. # M5005, Wisconsin, USA). The PCR primer pair, GRA6-FO and GRA6-RO, was designed from the GRA6 gene sequence and used in the primary PCR (Table 1). Two microliter of 1:10 diluted primary PCR product was used as a template in the secondary PCR using the internal primers described by Fazaeli et al. (2000), designated GRA6-F and GRA6-R (Table 1).

#### 2.4.5 Amplification of SAG2 locus

Genotyping of *T. gondii* was based on DNA polymorphisms at the SAG2 locus, encoding tachyzoite surface antigen p22. Samples were analyzed at the SAG2 locus using a nested-PCR approach that separately amplifies the 5′ and 3′ ends of the locus. The 5′ end of the SAG2 locus was amplified according to Howe et al. (1997) in two stages, namely the primary reaction using 20-mer forward primer (SAG2-F4) and a 20-mer reverse primer (SAG2-R4), and the secondary reaction using the forward primer (SAG2-F) and reverse primer (SAG2-R2) (Table 1). The reaction was run in a total of 50 μl, containing 200 μM of each of the four deoxynucleotide triphosphates (dNTP) (Promega, cat. # C1141, Wisconsin, USA), 25 pmol of each of primers SAG2-F4/R4 and SAG2-F/R2 (Sigma, Oakville, ON), 2.5 mM MgCl_2_ (Promega, cat. # A3513, Wisconsin, USA), 2.5 U of Go Taq Polymerase (Promega, cat. # M3005, Wisconsin, USA) and 1× PCR buffer (Promega, cat. # M791A, Wisconsin, USA). Two microliter of diluted DNA template (1/10 in DNase free water) were used in the primary PCR, whereas 5 μl of the first PCR product were used as template in the secondary PCR. The secondary PCR reagent concentrations were the same as those used in the primary PCR reaction. The 3′ end of the SAG2 locus was similarly analyzed with the primers SAG2-F3 and SAG2-R3 for the initial amplifications, and the internal primers SAG2-F2 and SAG2-R (Table 1) for the second round of amplification. Two microliter of diluted DNA template were used in the primary PCR, whereas 2 μl of the first PCR product were used as template in the secondary PCR. The primary and secondary PCR reagent concentrations and amplification conditions were the same as those used in the SAG2 5’ PCR. Primers were selected to separately amplify the 3′ and 5′ ends of the *T. gondii* SAG2 locus, resulting in 241-bp and 221-bp products respectively.

### 2.5 Genotyping by PCR-RFLP

RFLP analysis was done for the genes BTUB and GRA6 to confirm the genotype (i.e., type I, II or III). *T. gondii* DNA extracted from goose brains (type III) and from beluga (type II) were used as genotyping controls. PCR positive amplicons were incubated with the appropriate restriction enzymes (Table 1) according to the manufacturer's instructions (New England BioLabs Inc., Ipswich, MA). The digested PCR products were visualized by electrophoresis on 2.5% to 3.0% agarose gels containing GelRed.

### 2.6 DNA sequencing

PCR amplification products were purified using a Mini Elute PCR purification kit (Qiagen, Inc., Mississauga, ON) according to the manufacturer's protocol. The PCR product of *T. gondii* SAG2 amplification was subjected to bi-directional, automated sequencing (ABI PRISM BigDye Terminator v3.1 Cycle Sequencing Kit, Applied Biosystems, Foster City, CA) and sequenced in both directions using the same PCR primers used in the original amplifications. DNA sequences were aligned using Bioedit (version 7.0.9) and compared to known sequences in GenBank. Specifically, the DNA sequences derived from the BTUB and GRA6 gene PCR positive amplicons were compared with available sequences in GenBank representing *T. gondii* types II and III accession numbers AF249702 (Lehmann et al., 2000), MG588012 (Battisti et al., 2018), EF585682 (Lindstrom et al., 2008), KM246840 (Donahoe et al., 2014), GU325790 (Opsteegh et al., 2010), AB235427 (Zakimi et al., 2006) and KM372588 (Danehchin et al., 2016).

## 3 Results

Of the 281 packages of retail fresh meat examined, 12 (4.3%) were found to be positive for *T. gondii* DNA. Specifically, seven (7.5% of 94) were chicken breasts, three (3.2% of 94) were ground pork, and two (2.2% of 93) were ground beef (Table 2). With respect to individual chicken breasts, there were a total of nine (3.9% of 234) found to be positive (i.e., two of the packages contained more than one positive chicken breast), so the PCR prevalence of *T. gondii* in chicken breasts was very similar to that in pork and beef when individual chicken breasts were considered.

**Table 2:** Prevalence and genotypes of *Toxoplasma gondii* in retail fresh meat packages in Canada.

Retail meat	No. tested	No. positive (%)	No. type II (%)	No. type III (%)
Ground beef	93	2 (2.2)	1 (50.0)	1 (50.0)
Chicken breast	94	7 (7.5)^a^	5 (71.4)	2 (28.6)
Ground pork	94	3 (3.2)	2 (66.7)	1 (33.3)
Total	281	12 (4.3)	8 (66.7)	4 (33.3)

a Prevalence based on individual chicken breasts was 3.9% (9 of 234 samples).

A total of three meat samples were positive based on 529bp-PCR (two chicken and one beef), while six samples were positive based on B1-PCR (two chicken, three pork, and one beef). Three chicken samples were positive by PCR amplification of both genes.

The overall prevalence of *T. gondii* was very similar among the three provinces (Table 3). Only ground beef packages purchased in Ontario were positive for *T. gondii* (6.5%), and only ground pork packages purchased in British Columbia (6.3%) and Ontario (3.1%) were positive. Packages of chicken breasts purchased in all three provinces were positive for *T. gondii*.

**Table 3:** Prevalence of *Toxoplasma gondii* in retail fresh meat packages in Canada according to sampling location.

Location	Retail meat	No. tested	No. positive (%)
British Columbia	Ground beef	32	0 (0)
	Chicken breast	32	2 (6.3)
	Ground pork	32	2 (6.3)
	Total	96	4 (4.2)
Alberta	Ground beef	30	0 (0)
	Chicken breast	30	3 (10.0)
	Ground pork	30	0 (0)
	Total	90	3 (3.3)
Ontario	Ground beef	31	2 (6.5)
	Chicken breast	32	2 (6.3)
	Ground pork	32	1 (3.1)
	Total	95	5 (5.3)
	Grand Total	281	12 (4.3)

The overall *T. gondii* prevalence in all three meat types was higher in spring collection months, particularly during April and May, than in the summer or fall (Table 4, Table 5). This higher spring prevalence was seen in retail fresh meat from all three provinces (Table 4), and was particularly apparent in chicken breasts (Table 5). No positive samples of any type, nor from any province, were identified beyond early August.

**Table 4:** Prevalence of *Toxoplasma gondii* in retail fresh meat packages in Canada by sampling location (Apr.–Dec. 2015).

Week of	British Columbia		Alberta		Ontario			
	No. tested	No. positive (%)	No. tested	No. positive (%)	No. tested	No. positive (%)	Total no. tested	Total no. positive (%)
Apr. 6	3	0 (0)	3	0 (0)	3	0 (0)	9	0 (0)
Apr. 13	3	0 (0)	0	0	3	0 (0)	6	0 (0)
Apr. 20	3	1 (33.3)	3	1 (33.3)	3	1 (33.3)	9	3 (33.3)
Apr. 27	3	0 (0)	3	0 (0)	3	0 (0)	9	0 (0)
May 4	3	2 (66.7)	3	1 (33.3)	3	1 (33.3)	9	4 (44.4)
May 18	3	0 (0)	3	1 (33.3)	3	0 (0)	9	1 (11.1)
May 25	3	0 (0)	0	0	3	0 (0)	6	0 (0)
Jun. 1	3	0 (0)	3	0 (0)	3	0 (0)	9	0 (0)
Jun. 8	3	0 (0)	3	0 (0)	3	0 (0)	9	0 (0)
Jun. 15	3	0 (0)	3	0 (0)	3	1 (33.3)	9	1 (11.1)
Jun. 22	3	1 (33.3)	3	0 (0)	3	0 (0)	9	1 (11.1)
Jul. 6	3	0 (0)	3	0 (0)	3	0 (0)	9	0 (0)
Jul. 13	3	0 (0)	3	0 (0)	3	0 (0)	9	0 (0)
Jul. 20	3	0 (0)	3	0 (0)	3	1 (33.3)	9	1 (11.1)
Jul. 27	3	0 (0)	3	0 (0)	3	0 (0)	9	0 (0)
Aug. 3	3	0 (0)	3	0 (0)	3	1 (33.3)	9	1 (11.1)
Aug. 10	3	0 (0)	3	0 (0)	3	0 (0)	9	0 (0)
Aug. 17	3	0 (0)	3	0 (0)	3	0 (0)	9	0 (0)
Aug. 24	3	0 (0)	3	0 (0)	3	0 (0)	9	0 (0)
Sep. 7	3	0 (0)	3	0 (0)	3	0 (0)	9	0 (0)
Sep. 14	3	0 (0)	3	0 (0)	3	0 (0)	9	0 (0)
Sep. 21	3	0 (0)	6	0 (0)	3	0 (0)	12	0 (0)
Sep. 28	3	0 (0)	3	0 (0)	3	0 (0)	9	0 (0)
Oct. 5	3	0 (0)	0	0	3	0 (0)	6	0 (0)
Oct. 12	3	0 (0)	3	0 (0)	3	0 (0)	9	0 (0)
Oct. 19	3	0 (0)	3	0 (0)	3	0 (0)	9	0 (0)
Oct. 26	3	0 (0)	3	0 (0)	3	0 (0)	9	0 (0)
Nov. 2	3	0 (0)	3	0 (0)	3	0 (0)	9	0 (0)
Nov. 16	3	0 (0)	3	0 (0)	3	0 (0)	9	0 (0)
Nov. 23	3	0 (0)	3	0 (0)	2	0 (0)	8	0 (0)
Nov. 30	3	0 (0)	3	0 (0)	3	0 (0)	9	0 (0)
Dec. 7	3	0 (0)	3	0 (0)	3	0 (0)	9	0 (0)
	96	4 (4.2)	90	3 (3.3)	95	5 (5.3)	281	12 (4.3)

**Table 5:** Prevalence of *Toxoplasma gondii* in retail fresh meat packages in Canada by sample type (Apr.–Dec. 2015).

Week of	Ground beef		Chicken breast		Ground pork			
	No. tested	No. positive (%)	No. tested	No. positive (%)	No. tested	No. positive (%)	Total no. tested	Total no. positive (%)
Apr. 6	3	0 (0)	3	0 (0)	3	0 (0)	9	0 (0)
Apr. 13	2	0 (0)	2	0 (0)	2	0 (0)	6	0 (0)
Apr. 20	3	0 (0)	3	3 (100.0)	3	0 (0)	9	3 (33.3)
Apr. 27	3	0 (0)	3	0 (0)	3	0 (0)	9	0 (0)
May 4	3	0 (0)	3	2 (66.7)	3	2 (66.7)	9	4 (44.4)
May 18	3	0 (0)	3	1 (33.3)	3	0 (0)	9	1 (11.1)
May 25	2	0 (0)	2	0 (0)	2	0 (0)	6	0 (0)
Jun. 1	3	0 (0)	3	0 (0)	3	0 (0)	9	0 (0)
Jun. 8	3	0 (0)	3	0 (0)	3	0 (0)	9	0 (0)
Jun. 15	3	0 (0)	3	1 (33.3)	3	0 (0)	9	1 (11.1)
Jun. 22	3	0 (0)	3	0 (0)	3	1 (33.3)	9	1 (11.1)
Jul. 6	3	0 (0)	3	0 (0)	3	0 (0)	9	0 (0)
Jul. 13	3	0 (0)	3	0 (0)	3	0 (0)	9	0 (0)
Jul. 20	3	1 (33.3)	3	0 (0)	3	0 (0)	9	1 (11.1)
Jul. 27	3	0 (0)	3	0 (0)	3	0 (0)	9	0 (0)
Aug. 3	3	1 (33.3)	3	0 (0)	3	0 (0)	9	1 (11.1)
Aug. 10	3	0 (0)	3	0 (0)	3	0 (0)	9	0 (0)
Aug. 17	3	0 (0)	3	0 (0)	3	0 (0)	9	0 (0)
Aug. 24	3	0 (0)	3	0 (0)	3	0 (0)	9	0 (0)
Sep. 7	3	0 (0)	3	0 (0)	3	0 (0)	9	0 (0)
Sep. 14	3	0 (0)	3	0 (0)	3	0 (0)	9	0 (0)
Sep. 21	4	0 (0)	4	0 (0)	4	0 (0)	12	0 (0)
Sep. 28	3	0 (0)	3	0 (0)	3	0 (0)	9	0 (0)
Oct. 5	2	0 (0)	2	0 (0)	2	0 (0)	6	0 (0)
Oct. 12	3	0 (0)	3	0 (0)	3	0 (0)	9	0 (0)
Oct. 19	3	0 (0)	3	0 (0)	3	0 (0)	9	0 (0)
Oct. 26	3	0 (0)	3	0 (0)	3	0 (0)	9	0 (0)
Nov. 2	3	0 (0)	3	0 (0)	3	0 (0)	9	0 (0)
Nov. 16	3	0 (0)	3	0 (0)	3	0 (0)	9	0 (0)
Nov. 23	2	0 (0)	3	0 (0)	3	0 (0)	8	0 (0)
Nov. 30	3	0 (0)	3	0 (0)	3	0 (0)	9	0 (0)
Dec. 7	3	0 (0)	3	0 (0)	3	0 (0)	9	0 (0)
	93	2 (2.2)	94	7 (7.5)	94	3 (3.2)	281	12 (4.3)

Genotyping by means of PCR-RFLP and DNA sequencing demonstrated alleles of *T. gondii* types II and III in all three types of meat (Table 2). Nucleotide sequences revealed a high percentage of sequence similarity (99%) with published sequences of *T. gondii* BTUB and GRA6, thereby establishing their specificity. The SAG2 gene did not amplify well in the present study, and did not provide clear results by either RFLP analyses or DNA sequencing.

While *T. gondii* types II and III were present in equal numbers in ground beef, type II predominated in chicken breasts and ground pork. Overall, *T. gondii* type II predominated in the positive meat samples (66.7%), with type III being found in 33.3%. *T. gondii* types II and III were also found to be present in all three provinces sampled. In British Columbia, types II and III were each present in two meat samples, whereas in Alberta two samples showed type II and one had type III, and in Ontario, four samples had type II and one had type III. None of the meat samples tested demonstrated recombination of genotypes or co-infection of *T. gondii* genotypes.

## 4 Discussion

While surveillance studies have been done worldwide to determine the prevalence of *T. gondii* in retail meats (Reviewed in Guo et al., 2015), the present study represents the first large-scale surveillance study done in Canada. Results demonstrated a relatively low overall prevalence (4.3%) of *T. gondii* in packages of meat, which was similar to, or only slightly higher than, what has been reported in the U.S. and Mexico (Dubey et al., 2005; Galván-Ramirez et al., 2010), and considerably lower than that reported in the U.K. (Aspinall et al., 2002) and in numerous other countries (Guo et al., 2015). Some caution should be taken, however, in comparing the prevalence rates reported in these studies, as a variety of detection methods were used, and the tested meats in some studies were further processed.

While a small number of packages of all three types of fresh meats tested (i.e., beef, chicken and pork) were found to contain *T. gondii* DNA, chicken breasts showed a slightly higher prevalence than the other meat types when whole packages were considered. This was a somewhat surprising result given that chickens are considered to have a lower susceptibility to *T. gondii* than swine (Reviewed in Guo et al., 2015). The present findings are, however, supported by a recent study in the U.S. which demonstrated a relatively high seroprevalence in chickens and concluded that these animals are, in fact, important in the epidemiology of *T. gondii* (Ying et al., 2017). The higher prevalence in packages of chicken breasts than in the other meat types tested may be due, at least in part, to the fact that most packages contained three breasts, resulting in a greater amount of meat tested. The *T. gondii* prevalence in individual chicken breasts was comparable to that in the packages of ground beef and ground pork. These data suggest that further testing for *T. gondii* in chickens, and other meat animals, should be done in Canada in order to update the prevalence in these animals and their potential for food-borne transmission of *T. gondii*.

The prevalence of *T. gondii* in the present study was found to be very similar in all three sentinel sites (i.e., British Columbia, Alberta and Ontario). This is not altogether surprising as fresh meats in Canada are often distributed interprovincially. Another interesting finding, which is difficult to interpret, was that most of the positive PCR results, from all three provinces, were obtained during the first two months of sampling (April and May), and no positives were seen after the month of August. This higher springtime prevalence was most apparent in chicken breasts. Unfortunately, since there were no retail fresh meat samples collected from January to March, it was not possible to report on the prevalence earlier in the calendar year. As *T. gondii* tissue cysts remain viable in the tissues of intermediate hosts for long periods of time, this type of seasonality, as seen with enteric parasites, was unexpected. Further testing of meat samples collected earlier in the year (i.e., January to March) will be necessary to determine whether this higher *T. gondii* prevalence was specific to the springtime.

In the present study, PCR was used to determine the presence of *T. gondii* DNA, and the amplification of both the 529 bp and B1 genes was found to provide a more robust detection method. While PCR analysis provides rapid results, and allows for genotyping, it does not confirm the presence of intact parasites, nor does it differentiate between viable, infectious parasites and non-viable parasites. For this purpose, further testing using mouse and cat bioassays will need to be performed. However, as discussed by Aspinall et al. (2002), *T. gondii* tissue cysts are very robust and it is likely that at least some of the positive meat samples will have contained viable parasites.

The present study is the first to determine the genotypes of *T. gondii* present in retail meats in Canada. While only two genotyping markers (BTUB and GRA6) were used, several others are available and may be used in future studies to fully characterize the genotypes present. Alleles for both types II and III were found in the retail fresh meats from all provinces tested in this study, with type II predominating, especially in chicken and pork. Similarly, a large study in the U.S. demonstrated the presence of *T. gondii* types II and III in pork following cat bioassay, while all three genotypes were seen in isolates obtained from mice inoculated with faecal floats (Dubey et al., 2005). Types II and III, as well as a type II variant were also reported to be common in free-range chickens in the U.S. (Ying et al., 2017). Conversely, type I was reported to predominate in retail meats in the U.K. (Aspinall et al., 2002), Brazil (da Silva et al., 2005) and Iran (Fallah et al., 2011). While *T. gondii* type II is generally more common in human toxoplasmosis (Howe and Sibley, 1995; Howe et al., 1997; Ajzenberg et al., 2002), both types II and III are frequently reported in animals. The findings of the present study support an epidemiological association between meat consumption and human toxoplasmosis, however, the transmission patterns and relative virulence of the different *T. gondii* genotypes remain poorly understood.

Routine inspection programs for *T. gondii* tissue cysts in meat do not currently exist in most countries, including Canada. There are too few tissue cysts found in infected food animals to detect by means of histological examination (Dubey et al., 1986a) and large-scale serological testing is not considered feasible. Until such time as the prevalence of *T. gondii* in food animals can be reduced considerably, it will be impractical for meat inspection programs to eliminate toxoplasmosis by requiring condemnation of infected animals (Reviewed in Leighty, 1990).

Treatment of retail fresh meats by cooking or freezing is an effective means of killing any viable *T. gondii* which may be present, rendering the meat safe for consumption (Reviewed in Jones and Dubey, 2012). While there is some dispute as to the exact temperatures required to destroy *T. gondii* tissue cysts in meat, Dubey et al. (1990) examined the effective time of exposure at different temperatures. Tissue cysts remained viable at 52 °C for 9.5 min but not at 58 °C for the same period. Viability could also generally be destroyed at 61 °C for 3.6 min. Dubey (1988) found that tissue cysts in pork were rendered non-viable after freezing at −12 °C for 3 days. Similarly, a temperature of −12.4 °C was obtained as the theoretical point at which *T. gondii* would be instantaneously inactivated (Kotula et al., 1991). Other treatments, including irradiation (Dubey et al., 1986b) and high pressure processing (Lindsay et al., 2006), have also been shown to be effective in inactivating tissue cysts.

Finally, pumping of pork products with salt solutions containing 2% NaCl or ≥1.4% potassium or sodium lactate was shown to inactivate *T. gondii* tissue cysts in pork (Hill et al., 2004), and Jones and Dubey (2012) noted that, in the U.S., much of the pork and chicken sold is injected with salts and water. However, the effects of these types of treatments on the viability of *T. gondii* in meat have not been determined critically (Bayarri et al., 2012), and they should not be used exclusively for destroying tissue cysts.

## 5 Conclusions

The present study demonstrated a relatively low overall prevalence of *T. gondii* in retail fresh meats in Canada, which was very similar among the meat types tested, and among the provinces sampled. There was a predominance of *T. gondii* type II in the meats tested. The findings of the present study fill an important knowledge gap in Canada by providing baseline data, which may allow for more accurate health risk assessments for the purpose of developing food safety guidelines and policies. As it was based only on PCR testing, with no indication of parasite viability, the prevalence of *T. gondii* in retail fresh meats reported in the present study may not be a true representation of the risk to consumers. Further testing involving bioassays will be necessary to estimate the viability and infectivity of *T. gondii* in retail fresh meats. The risk of meat-borne transmission of this parasite can be minimized at the consumer level by means of thorough cooking or freezing.
